# The Risk of Stable Partnerships: Associations between Partnership Characteristics and Unprotected Anal Intercourse among Men Who Have Sex with Men and Transgender Women Recently Diagnosed with HIV and/or STI in Lima, Peru

**DOI:** 10.1371/journal.pone.0102894

**Published:** 2014-07-16

**Authors:** Mary C. Cambou, Amaya G. Perez-Brumer, Eddy R. Segura, H. Javier Salvatierra, Javier R. Lama, Jorge Sanchez, Jesse L. Clark

**Affiliations:** 1 David Geffen School of Medicine at UCLA, Department of Medicine, Division of Infectious Diseases and Program in Global Health, Los Angeles, California, United States of America; 2 Mailman School of Public Health, Columbia University, Department of Sociomedical Sciences, New York, New York, United States of America; 3 Asociación Civil Impacta Salud y Educación, Lima, Peru; The University of New South Wales, Australia

## Abstract

**Background:**

Partnership type is an important factor associated with unprotected anal intercourse (UAI) and subsequent risk for HIV and sexually transmitted infections (STI). We examined the association of partnership type with UAI among men who have sex with men (MSM) and male-to-female transgender women (TGW) in Lima, Peru, recently diagnosed with HIV and/or STI.

**Methods:**

We report data from a cross-sectional analysis of MSM and TGW recently diagnosed with HIV and/or STI in Lima, Peru between 2011 and 2012. We surveyed participants regarding UAI with up to their three most recent sexual partners according to partner type. Multivariable Generalized Estimate Equating (GEE) models with Poisson distribution were used to estimate prevalence ratios (PR) for UAI according to partner type.

**Results:**

Among 339 MSM and TGW recently diagnosed with HIV and/or STI (mean age: 30.6 years, SD 9.0), 65.5% self-identified as homosexual/gay, 16.0% as bisexual, 15.2% as male-to-female transgender, and 3.3% as heterosexual. Participants provided information on 893 recent male or TGW partners with whom they had engaged in insertive or receptive anal intercourse: 28.9% stable partners, 56.4% non-stable/non-transactional partners (i.e. casual or anonymous), and 14.7% transactional partners (i.e. transactional sex client or sex worker). Unprotected anal intercourse was reported with 41.3% of all partners. In multivariable analysis, factors associated with UAI included partnership type (non-stable/non-transactional partner APR 0.73, [95% CI 0.59–0.91], transactional partner APR 0.53 [0.36–0.78], p<0.05) and the number of previous sexual encounters with the partner (>10 encounters APR 1.43 [1.06–1.92], p<0.05).

**Conclusion:**

UAI was more commonly reported for stable partners and in partnerships with >10 sexual encounters, suggesting UAI is more prevalent in partnerships with a greater degree of interpersonal commitment. Further research assessing partner-level factors and behavior is critical for improving HIV and/or STI prevention efforts among Peruvian MSM and TGW.

## Introduction

The HIV epidemic in Peru is concentrated among men who have sex with men (MSM) and male-to-female transgender women (TGW), with estimates of HIV prevalence among MSM ranging from 11.0 to 18.5% [1], [2] and as high as 30% among TGW [3]. The frequency of unprotected anal intercourse (UAI) remains high among these populations, underscoring the need to better assess factors influencing condom use [4]. In a prior surveillance study of sexual risk behavior among MSM in Lima, Peru, only 54.4% of HIV-uninfected and 48.4% HIV-infected MSM reported condom use with their last male sex partner [2]. Similarly high rates of UAI with serodiscordant or unknown HIV status partners have been reported in other research with MSM and TGW populations in Peru [5], [6].

Previous research has suggested an association between partner type and sexual risk behavior in MSM and TGW partnerships [1], [7]–[16]. In general, “stable” or primary partnerships are associated with a greater perception of commitment between the partners involved, while “non-stable” partnerships, including casual, anonymous or transactional sexual relations, typically do not carry comparable levels of emotional intimacy [14]–[17]. Accordingly, UAI has been more commonly reported in primary partnerships when compared to casual partnerships among both HIV-infected and HIV-uninfected MSM [10], [11], [18], suggesting an association between partnership type and willingness to engage in UAI. The greater intimacy presumed in “stable” partnerships can be seen as both protective (supporting open communication about HIV and STI, and encouraging mutually protective behavior within the partnership), and potentially harmful (inhibiting condom use within a partnership which is incorrectly believed to be monogamous and free of infection) [14]–[17].

While previous research has documented an association between partnership type and UAI in diverse populations worldwide [7], [10], [12], [19], [20], few studies have investigated the relationship between partnership type and UAI among MSM and TGW in Latin America [11], [21]. Modeling estimates from the Prevention Umbrella for MSM in the Americas (PUMA) project have suggested that UAI drives the HIV epidemic in Peru, with approximately one-third of new HIV cases believed to occur in primary partnerships [1]. Other studies evaluating the potential use of harm-reduction techniques among HIV-infected and HIV-uninfected MSM and TGW in Peru have found no evidence of serosorting, discussion of HIV infection status, or other partner-specific strategies to control risk of HIV/STI transmission [4], [6]. Epidemiologic studies from across Latin America have reported a higher overall prevalence of UAI with stable compared with casual partners among MSM and TGW, though we are not aware of any studies in the region that have conducted partner-level analyses of the association between UAI and partner type [2], [3], [22], [23]. Detailed knowledge of how partner type influences sexual risk behavior among MSM and TGW in Latin America will contribute to the development of culturally- and epidemiologically-specific interventions to control the spread of HIV and STIs in this population.

To address this gap in knowledge, we assessed the association between partnership type and UAI among up to the three most recent partners of MSM and TGW recently diagnosed with HIV and/or STI in Lima, Peru. Understanding the dynamics of UAI in relation to partnership structure may play an integral role in the development of and implementation of culturally-appropriate HIV prevention efforts for at-risk MSM and TGW. By focusing specifically on individuals with a recent HIV and/or STI diagnosis, we are able to address a unique subgroup of MSM and TGW with biologically confirmed exposure to HIV and/or STI and to assess their risk for further disease transmission within their sexual networks.

## Materials and Methods

### Data Collection

We conducted a secondary analysis of partnership characteristics associated with recent UAI among MSM and TGW recently diagnosed with HIV and/or STI as part of a survey of partner notification beliefs and practices among MSM and TGW in Peru. Between January, 2011 and January, 2012, we enrolled 397 MSM and TGW diagnosed with HIV and/or STI within the previous 30 days. MSM and TGW ≥18 years of age and diagnosed with HIV and/or STI as part of routine care at the Asociación Civil Impacta Salud y Educación clinical research unit or the Alberto Barton municipal STI clinic, both in Lima-Callao, were referred for study enrollment by clinic staff. Enrollment was limited to men or TGW who reported anal or oral intercourse with a male or TGW partner during the previous year, and who had been diagnosed with HIV, syphilis, genital herpes and/or gonorrhea/chlamydia (GC/CT) within the previous 30 days. After completing post-test counseling (including standard partner notification recommendations), participants were invited to complete a survey which explored attitudes, beliefs, and anticipated practices related to partner notification. In order to accommodate potential emotional distress following HIV and/or STI diagnosis, participants were allowed to complete the survey either immediately after post-test counseling or at a subsequent appointment scheduled within 30 days.

Participants were asked to provide information on demographic information, sexual identity, and sexual practices (both in general over the preceding three months and specifically with each of their last three partners). Data on participants’ aggregate sexual practices during the previous three months, including engagement in transactional sex, was collected. Participants were also asked to describe the characteristics of their three most recent partners, including participants’ perception of their partner’s gender, sexual orientation, and sexual role during intercourse (“*activo*” or insertive, “*pasivo*” or receptive, “*moderno*” or versatile), as well as the partner type (primary/stable, casual, anonymous, transactional sex client, transactional sex worker). Participants were also asked to describe the specific sexual practices performed with each of their three most recent partners, including act-specific condom use, and their history of prior sexual encounters with the partner. Participants were compensated 10 *Nuevos soles* (approximately $4 USD) for their transportation costs.

### Statistical Analysis

For this analysis, inclusion was limited to participants who reported only male or male-to-female TGW as their most recent sexual partners (ranging from one partner over a lifetime to the three most recent partners), and who reported engaging in anal intercourse (receptive or insertive) with all reported partners. The main outcome (UAI) was constructed at the partnership level as a dichotomous variable, where the outcome for each partnership reported was defined as engagement in insertive and/or receptive UAI. Partner type was defined by participant report, and limited to the following categories: stable, casual, anonymous, transactional sex client or transactional sex worker. Other variables assessed included participant age, education, sexual identity, sexual role, and involvement in transactional sex within the last three months, as well as partner sexual identity and sexual role, type of partnership, and number of sexual encounters with the partner (1, 2 to 3, 4 to 10 or >10).

Descriptive analyses were conducted for each participant and their three most recent partners. We tested associations between independent variables (at both participant and partner-levels) and the primary UAI outcome using chi-square and Student’s t-tests. For the bivariate and multivariable analysis, we redefined “casual partners” and “anonymous partners” as “non-transactional/non-stable partners”, and redefined “transactional sex worker partners” and “transactional sex client partners” as “transactional sex partners.” Variables associated with the outcome at a p-value<0.20 in bivariate analysis were considered for the multivariable regression model, an approach that performs better than when traditional, lower cut-off values are used [24]. To measure the association between the independent variables and the outcomes, we computed prevalence ratios (PR) by using Poisson regression analyses with robust estimation of standard errors [25]–[27]. Moreover, we used its Generalized Estimating Equations (GEE) extension [28] with an exchangeable working correlation matrix to account for the correlation between partner-level data reported by the same participant (maximum of three partners reported). We used Stata 12.0 (Stata Corp, College Station, TX, US) in all analyses.

### The Ethics Statement

The study was approved by the Institutional Review Boards of the University of California, Los Angeles (G10-03-036-01), and Asociación Civil Impacta Salud y Educación, Peru (0104-2010-CE). Written informed consent was obtained from all participants prior to enrollment and initiating any study procedures.

## Results

### Study Population

We surveyed 397 MSM and TGW with a recent HIV and/or STI diagnosis, of whom 339 met inclusion criteria for this analysis (insertive or receptive anal intercourse with male or male-to-female TGW partners in each of their three most recent sexual partnerships). Table 1 describes the demographic and behavioral characteristics of participants included in the analysis. The mean age was 30.6 years (Range = 18–60 years; SD = 9.0 years), and 78.2% reported completion of high school or an education beyond high school. For HIV and/or STI diagnosis (syphilis, genital herpes, and/or GC/CT), 53.4% reported a non-HIV STI, 23.0% reported HIV only, and 23.6% reported HIV plus another STI. For sexual orientation/gender identity, 65.5% self-identified as homosexual/gay, 16.0% as bisexual, 15.2% as transgender and 3.3% heterosexual. Involvement in transactional sex during the last three months was reported by 31.9% of participants: 94/108 reported having at least one partner within the last three months who paid or gave gifts to the participant in exchange for sex, 9/108 reported having at least one partner within the last three months to whom the participant paid or gave gifts in exchange for sex, and 5/108 reported having both types of partners. Greater than 2/3 of the study population (67.8%) reported on their three most recent partners, while 17.4% reported on two partners over their lifetime, and 14.7% reported on one partner over their lifetime.

**Table 1:** Characteristics of MSM and TGW recently diagnosed with HIV and/or STI reporting exclusively male or TGW partners; Lima, Peru 2011–2012.

	N = 339*
Characteristics	n (%)
**Age (Years) Mean; SD**	30.6; 9.0
**Education**	
Less than High School	74 (21.8)
Completed High School	125 (36.9)
Higher Education (University, Tech)	140 (41.3)
**HIV and/or STI Diagnosis** †	
Non-HIV STI	181 (53.4)
HIV Only	78 (23.0)
HIV plus Another STI	80 (23.6)
**Sexual Orientation/Gender Identity**	
Heterosexual	11 (3.3)
Bisexual	54 (16.0)
Homosexual	220 (65.5)
Transgender	51 (15.2)
**Sexual Role During Intercourse**	
*Activo* (Insertive)	46 (13.7)
*Pasivo* (Receptive)	131 (39.0)
*Moderno* (Versatile)	159 (47.3)
**Engaged in Transactional Sex Within Last 3 Months**	108 (32.4)
**Number of Partnerships Reported**	
3 Partners	230 (67.8)
2 Partners	59 (17.4)
1 Partner	50 (14.8)

*Totals for some variables do not add up to 339 due to missing data.

† Report of HIV, syphilis, genital herpes and/or gonorrhea/chlamydia (GC/CT) within the previous 30 days.

Characteristics of recent male and TGW partners (N = 893) are described in Table 2. Participants described 52.3% of their recent male partners’ sexual role as “*activo*” (insertive), 32.1% as “*moderno*” (versatile sexual role), and 15.6% as “*pasivo*” (receptive). Based on our reclassification of partner types, 28.9% of partners were described as stable, 56.4% as non-transactional/non-stable, and 14.7% as transactional. Overall, among the 893 recent partnerships where anal intercourse was reported, 41.3% involved UAI.

**Table 2:** Characteristics of recent male or TGW partners among MSM and TGW recently diagnosed with HIV and/or STI; Lima, Peru 2011–2012.

	N = 893*
	n (%)
Characteristics	
**Perceived Partner Sexual Orientation/Gender Identity**	
Heterosexual	115 (13.8)
Bisexual	342 (40.9)
Homosexual	354 (42.4)
Transgender	24 (2.9)
**Perceived Partner Sexual Role During Intercourse**	
*Activo* (Insertive)	463 (52.3)
*Pasivo* (Receptive)	138 (15.6)
*Moderno* (Versatile)	285 (32.1)
**Partner Type**	
Stable	255 (28.9)
Casual	398 (45.1)
Anonymous	100 (11.3)
Sex Client	123 (13.9)
Sex Worker	7 (0.8)
**UAI within Partnership** †	337 (41.3)
**Frequency of Sexual Encounters with Reported Partner**	
1	262 (29.5)
2 to 3	221 (25.0)
4 to 10	166 (18.7)
>10	238 (26.8)

*Totals for all variables do not add up to 893 due to missing data.

† Percentage calculated from n = 817, for the subset of partnerships analyzed in the bivariate and multivariable models.

### Sexual Risk Behavior

Univariate analyses of associations between UAI with participant-level and partner-level characteristics (using cluster-adjusted linear regression for age and cluster-adjusted chi-square tests for categorical variables) are described in Table 3. In the univariate analysis, only partnership type and number of previous sexual encounters with the partner were significantly associated with UAI (p<0.05). Neither participants’ or their partners’ sexual identity or sexual role, nor engagement in transactional sex within the last three months were significantly associated with prevalence of UAI.

**Table 3:** Characteristics Associated with Unprotected Anal Intercourse in Recent Partnerships of MSM and TGW Diagnosed with HIV and/or STI; Lima, Peru 2011–2012.

	UAI with Partner (n = 337)	No UAI with Partner (n = 480)	p
	n (%)	n (%)	
Characteristics			
**Age (Years) Mean; SD**	31.0; 12.1	30.3; 14.5	0.36
**Education**			
Less than Complete Secondary School Education	77 (41.2)	110 (58.8)	0.97
Secondary School Graduate	120 (42.0)	166 (58.0)	
Higher education (University, Technical Institute, etc.)	140 (40.7)	204 (59.3)	
**Participant Sexual Orientation/Gender Identity**			
Heterosexual	5 (35.7)	9 (64.3)	0.49
Bisexual	56 (49.1)	58 (50.9)	
Homosexual	216 (39.6)	329 (60.4)	
Transgender	57 (41.9)	79 (58.1)	
**Participant Sexual Role During Intercourse**			
*Activo* (Insertive)	41 (48.2)	44 (51.8)	0.56
*Pasivo* (Receptive)	143 (41.2)	204 (58.8)	
*Moderno* (Versatile)	153 (39.9)	230 (60.1)	
**Transactional Sex Within Last 3 Months**			
Yes	103 (36.3)	181 (63.7)	0.15
No	224 (43.2)	294 (57.8)	
**Perceived Partner Sexual Orientation/Gender Identity**			
Heterosexual	46 (43.8)	59 (56.2)	0.61
Bisexual	135 (43.1)	178 (56.9)	
Homosexual	124 (38.3)	200 (61.7)	
Transgender	9 (50.0)	9 (50.0)	
**Perceived Partner Sexual Role During Intercourse**			
*Activo* (Insertive)	182 (42.5)	246 (57.5)	0.74
*Pasivo* (Receptive)	46 (38.0)	75 (62.0)	
*Moderno* (Versatile)	105 (40.5)	154 (59.5)	
**Partner Type**			
Stable	139 (60.7)	90 (39.3)	**<0.05**
Non-Stable/Non-Transactional (Casual or Anonymous)	157 (34.4)	299 (65.6)	
Transactional (Sex Client or Sex Worker)	35 (28.9)	86 (71.1)	
**Number of Previous Sexual Encounters with Partner**			
1	75 (32.1)	159 (76.9)	**<0.05**
2 to 3	72 (35.8)	129 (64.2)	
4 to 10	67 (42.4)	91 (57.6)	
>10	120 (55.1)	98 (44.9)	

Table 4 describes the crude and adjusted PR for factors associated with UAI, as estimated by Poisson regression analysis with a GEE extension. In the bivariate analysis, the following factors were associated with UAI: partnership type (non-stable/non-transactional sexual partners: PR 0.62, [95% CI 0.52–0.74] and transactional sex partners: PR 0.48 [95% CI 0.33–0.69] reference: stable partners, p<0.05), and number of previous sexual encounters with the partner (4–10 encounters: PR 1.41 [95% CI 1.06–1.87], >10 encounters: PR 1.80 [95% CI 1.39–2.34], reference: 1 encounter, p<0.05). In the multivariable analysis, both partnership type and number of sexual encounters were significantly associated with UAI, with a slight increase in the PR estimates for non-stable/non-transactional partners (PR 0.73 [95% CI 0.59–0.91], p<0.05) and transactional sex partners (PR 0.53 [95% CI 0.36–0.78] p<0.05), and a slight decrease in in the PR estimate for >10 encounters (PR 1.43 [95% CI 1.06–1.92], p<0.05). Participant age, education, sexual identity, sexual role, recent history of transactional sex, as well as partner sexual identity and sexual role were not significantly associated with UAI in the multivariable model.

**Table 4:** Crude and adjusted prevalence ratios (PR) of participant and partner-level characteristics associated with unprotected anal intercourse in recent partnerships of MSM and TGW diagnosed with HIV and/or STI; Lima, Peru 2011–2012.

	Crude PR(n = 817)	95% CI	p	Adjusted PR(n = 787)	95% CI	p
Characteristics						
**Age (Years)**	0.99	0.98–1.01	0.56			
**Education**						
Less Than Complete Secondary School Education	1	Ref	-			
Secondary School Graduate	1.01	0.76–1.34	0.95			
Higher Education (University, Technical Institute, etc.)	1.02	0.77–1.34	0.9			
**Sexual Orientation/Gender Identity**						
Heterosexual	1	Ref	-			
Bisexual	1.44	0.65–3.18	0.37			
Homosexual	1.13	0.52–2.44	0.76			
Transgender	1.17	0.52–2.63	0.7			
**Sexual Role During Intercourse**						
*Activo* (Insertive)	1	Ref	-			
*Pasivo* (Receptive)	0.84	0.61–1.14	0.26			
*Moderno* (Versatile)	0.83	0.61–1.12	0.22			
**Transactional Sex Within Last 3 Months**						
Yes	1	Ref	-	1	Ref	-
No	0.82	0.65–1.04	0.1	0.99	0.78–1.24	0.9
**Perceived Partner Sexual Orientation/Gender Identity**						
Heterosexual	1	Ref	-			
Bisexual	1.04	0.79–1.37	0.79			
Homosexual	1	0.74–1.35	0.98			
Transgender	1.13	0.68–1.87	0.63			
**Perceived Partner Sexual Role During Intercourse**						
*Activo* (Insertive)	1	Ref	-			
*Pasivo* (Receptive)	0.94	0.70–1.24	0.64			
*Moderno* (Versatile)	0.99	0.81–1.20	0.9			
**Partnership Type**						
Stable	1	Ref	-	1	Ref	-
Non-Stable/Non-Transactional (Casual or Anonymous)	0.62	0.52–0.74	**<0.05**	0.73	0.59–0.91	**<0.05**
Transactional (Sex Client or Sex Worker)	0.48	0.33–0.69	**<0.05**	0.53	0.36–0.78	**<0.05**
**Frequency of Sexual Encounters with Reported Partner**						
1	1	Ref	-	1	Ref	-
2 to 3	1.25	0.95–1.65	0.11	1.11	0.84–1.48	0.47
4 to 10	1.41	1.06–1.87	**<0.05**	1.21	0.91–1.62	0.2
>10	1.8	1.39–2.34	**<0.05**	1.43	1.06–1.92	**<0.05**

## Discussion

In our analysis of Peruvian MSM and TGW with a new HIV and/or STI diagnosis, the prevalence of UAI in recent sexual partners was greatest in stable partnerships with a history of more than 10 previous episodes of sexual. These findings may aid in the development and refinement of culturally-specific HIV and/or STI prevention interventions for MSM and TGW in Latin America, particularly interventions directed towards reducing risk of transmission within MSM and TGW partnerships and networks. The high frequency of UAI reported during partnerships characterized as “stable” or “primary,” as well as in partnerships with a high number of sexual contacts, suggests that the greater sense of familiarity, commitment, and/or intimacy common in these relationships may lead to a minimization of the importance of condom use during anal intercourse.

The fact that a large percentage (46.6%) of this sample of Peruvian MSM and TGW was recently diagnosed with HIV underscores the potential risk of a “stable” partnership by questioning implied sexual fidelity, and emphasizing the high level of transmission risk faced by both these individuals and their partners within their stable partnerships. Previous studies from the U.S. and Europe have addressed the use of serosorting, seropositioning, and other forms of “negotiated safety” to reduce the risk of HIV and/or STI transmission during UAI within stable partnerships [29]–[32]. We are not aware of any research conducted among MSM or TGW in Latin America that specifically addresses these harm reduction strategies, although epidemiologic studies conducted in Peru have found no difference in the frequency of reported UAI when analyzed according to the HIV status of MSM and TGW surveyed and/or their partners [4]. Similarly, a recent analysis of Peruvian national surveillance data found that the prevalence of UAI was high in both seroconcordant and serodiscordant partnerships, though HIV serostatus was never discussed in the vast majority of recent partnerships involving UAI [6]. These results highlight the need for further research to explore the potential impact of interventions designed to promote knowledge and discussion of partner HIV status among MSM and TGW in Latin America within different partnerships contexts.

Literature on potential associations between partnership characteristics (such as partnership type and frequency of sexual encounters) and risks for HIV and/or STI among MSM and TGW networks in Peru is scarce, and prevention strategies that acknowledge differences in HIV and/or STI risk according to partnership type are underdeveloped. We are currently conducting ongoing research in Lima to explore how beliefs and attitudes regarding HIV and/or STI vary between different partnership types, including how partnership structures influence engagement in UAI, and how partnership patterns impact the spread of HIV and/or STI through MSM and TGW sexual networks [33]. A more detailed understanding of how partnership formations influence HIV and/or STI risk will contribute to the development of combination HIV prevention approaches incorporating different prevention technologies within distinct partnership contexts [34], [35].

Our findings suggest that an important challenge for future interventions will be to address whether and how assumptions of trust, commitment, monogamy, and/or fidelity within stable partnerships influence decisions regarding condom use and other prevention technologies. In partnerships with open, direct communication about HIV and/or STI risk and sexual behavior, prevention strategies including routine counseling and testing, serostatus disclosure, and partner notification and treatment (following HIV and/or STI diagnosis) are more likely to be effective. In seroconcordant, HIV-uninfected stable partnerships, negotiated safety contracts might also aid in reducing HIV and/or STI transmission despite regular UAI, though studies demonstrate mixed results with negotiated safety as a risk reduction strategy. As a result, inconsistent condom use outside of the primary relationship may lead to increased risk of HIV and or/STI exposure within the partnership and should be used cautiously as a potential prevention strategy [36], [37].

In contrast, for casual or anonymous partnerships, or for stable partnerships where interpersonal commitment and communication are limited, routine use of self-protective techniques including condom use, Pre-Exposure Prophylaxis (if HIV-uninfected) or antiretroviral therapy (if HIV-infected), and potentially rectal microbicides could also be used to reduce the risk of HIV transmission [38]. Given the complexity of partnership frameworks of MSM and TGW in Peru and the high risk of HIV and/or STI acquisition within their sexual networks, it is likely that any effective prevention strategy will need to incorporate multiple complementary prevention techniques. In this context, our findings provide preliminary data to guide the development of partner-specific prevention strategies for MSM and TGW in Peru.

Our analysis has strengths and limitations. Although we provided specific descriptions of different partner types in our survey, variations in personal definitions of partnership type are common, and the interpretation of what constitutes a “stable” versus a “casual” or even “commercial” partner might not be uniform across our study participants. We also did not collect information concerning sexual exclusivity or partner serostatus within reported partnerships, factors that may also be involved in decisions about whether or not to engage in UAI with a given partner. Participants in our study were limited to individuals diagnosed with an STI within the last 30 days, a group that is important for understanding behavioral factors leading to actual STI acquisition, but one that is also more likely to have engaged in recent sexual risk behavior and not necessarily representative of all MSM and TGW in Peru. However, the high prevalence of both sexual risk behavior and HIV and/or STI observed in our sample is comparable with other published data on MSM and TGW in Peru, suggesting consistency with other studies on risk behavior and disease transmission among Peruvian MSM and TGW [2], [39]. Despite these limitations, our analysis provides important information regarding the association between UAI and partnership characteristics among recently-infected MSM and TGW in Peru.

Our findings indicate the need for further investigation on how partner type and other partnership characteristics influence sexual risk behavior and HIV and/or STI transmission among MSM and TGW. In contrast to previous studies that have assessed behavior with only the last partner and/or more general patterns of recent sexual risk behavior, our study provides a broader view of partnership-level risk factors by describing individual histories of sexual risk behavior with recent sexual partners. By placing partner-level factors at the center of a multi-component HIV and/or STI prevention strategy, researchers and public health officials may begin to better address the diverse range of risk contexts potentiating the spread of HIV and/or STI in MSM and TGW populations in Lima, Peru.
